# Effects of White Matter Injury on Resting State fMRI Measures in Prematurely Born Infants

**DOI:** 10.1371/journal.pone.0068098

**Published:** 2013-07-09

**Authors:** Christopher D. Smyser, Abraham Z. Snyder, Joshua S. Shimony, Tyler M. Blazey, Terrie E. Inder, Jeffrey J. Neil

**Affiliations:** 1 Department of Neurology, Washington University, Saint Louis, Missouri, United States of America; 2 Department of Pediatrics, Washington University, Saint Louis, Missouri, United States of America; 3 Mallinckrodt Institute of Radiology, Washington University, Saint Louis, Missouri, United States of America; Institution of Automation, CAS, China

## Abstract

The cerebral white matter is vulnerable to injury in very preterm infants (born prior to 30 weeks gestation), resulting in a spectrum of lesions. These range from severe forms, including cystic periventricular leukomalacia and periventricular hemorrhagic infarction, to minor focal punctate lesions. Moderate to severe white matter injury in preterm infants has been shown to predict later neurodevelopmental disability, although outcomes can vary widely in infants with qualitatively comparable lesions. Resting state functional connectivity magnetic resonance imaging has been increasingly utilized in neurodevelopmental investigations and may provide complementary information regarding the impact of white matter injury on the developing brain. We performed resting state functional connectivity magnetic resonance imaging at term equivalent postmenstrual age in fourteen preterm infants with moderate to severe white matter injury secondary to periventricular hemorrhagic infarction. In these subjects, resting state networks were identifiable throughout the brain. Patterns of aberrant functional connectivity were observed and depended upon injury severity. Comparisons were performed against data obtained from prematurely-born infants with mild white matter injury and healthy, term-born infants and demonstrated group differences. These results reveal structural-functional correlates of preterm white matter injury and carry implications for future investigations of neurodevelopmental disability.

## Introduction

Survival rates for prematurely-born infants have dramatically improved following advances in perinatal and neonatal care. However, brain injury in this population remains common. Approximately 7–23% of very low birth weight infants develop intraventricular hemorrhage (IVH), and up to 50% sustain cerebral white matter injury (WMI) [1]–[4]. WMI can range from severe forms, including cystic periventricular leukomalacia (cPVL) and periventricular hemorrhagic infarction (PHI), to mild forms such as focal punctate white matter lesions. WMI is often associated with more widespread cerebral lesions involving the cortical and deep gray matter [3]–[5]. This correlation between abnormalities of white and gray matter may result from concurrent primary insult or secondary deafferentiation of neurons following axonal degeneration. Regardless of mechanism, moderate-severe WMI remains a major risk factor for subsequent functional impairment in motor and cognitive domains in prematurely-born infants.

Conventional magnetic resonance imaging (MRI) has high sensitivity for delineating the patterns of WMI related to neurodevelopmental outcome that are common in preterm infants [6]–[10]. In addition, diffusion tensor imaging (DTI) has been used to characterize the microstructural consequences of WMI [11], [12], and these effects have been related to brain development [13] and neurodevelopmental outcome [14]–[19]. Although these MR techniques are better predictors of outcome than other clinical or imaging measures, their predictive power remains limited, perhaps owing to the complex relationship between cerebral structure and function.

In contrast to structural MRI, resting state functional connectivity MRI (rs-fcMRI) indirectly measures neural activity by assessing spontaneous, low-frequency fluctuations in blood oxygen level dependent (BOLD) signal [20]–[24]. These fluctuations are temporally coherent within widely distributed parts of the brain that together constitute resting state networks (RSNs). The importance of RSNs lies in the fact that their spatial topography recapitulates fMRI responses to a wide variety of cognitive, motor and sensory tasks [23], [25], [26]. Recent investigations have established the utility of rs-fcMRI in the study of typical and atypical neurodevelopment [27]–[31]. Similarly, rs-fcMRI has been used to study the impact of WMI on RSN development in childhood and early adulthood [32]–[34].

As a technique, rs-fcMRI has features that render it suitable for studying infants, including i) ascertainment of global connectivity properties in minutes; ii) no requirement for task performance during acquisition; and iii) the availability of rs-fcMRI data sets for preterm and term-born infants without cerebral injury that provide a reference for comparison [35]–[41]. Thus far, there have been no studies on the impact of moderate-severe WMI on prematurely-born infants during early development using rs-fcMRI. Here, we report results establishing the feasibility of using this modality to study this population.

## Methods

### Subjects

Infants were prospectively recruited from the St. Louis Children’s Hospital Neonatal Intensive Care Unit (NICU) during the period from 2007–2012. WMI subjects were identified based upon results of head ultrasound studies routinely obtained in very preterm infants (born prior to 30 weeks gestation) by the clinical teams. Infants with periventricular echodensity persisting for greater than seven days were recruited into the WMI group. Prematurely-born infants without cranial ultrasound abnormalities and healthy, term-born control subjects were also recruited.

#### Ethics statement

All aspects of the study were approved by the Washington University School of Medicine’s Human Studies Committee. Parental informed, written consent was obtained for each subject prior to participation in the study.

### Data Acquisition

All subjects underwent MRI at term-equivalent postmenstrual age (PMA). Infants were studied during natural sleep or while resting quietly without sedation [42]. Noise protection during scans was provided through use of ear muffs (Natus Medical, Foster City, CA). Arterial oxygen saturation and heart rate were continuously monitored throughout the session. Images were acquired on a Siemens Magnetom Trio 3T scanner (Erlangen, Germany) using an infant-specific head coil (Advanced Imaging Research, Cleveland, OH). Structural images were collected using a turbo-spin echo (TSE), T2-weighted sequence (TR 8600 ms; TE 160 ms; voxel size 1×1×1 mm^3^; echo train length 17 ms). Functional images were collected utilizing a single shot, gradient echo, echo-planar-image (EPI) T2*-weighted sequence sensitized to BOLD signal changes (TR 2910 ms; TE 28 ms; voxel size 2.4×2.4×2.4 mm^3^; flip angle 90°; FOV 151 mm). Whole brain coverage was obtained with 44 contiguous slices. A minimum of 200 whole-brain volumes (frames) was obtained for each subject. Additional fMRI data were obtained in a limited number of participants depending upon subject tolerance. All MRI data are available at the Washington University Central Neuroimaging Data Archive (CNDA) (https://cnda.wustl.edu).

### Data Analysis

#### WMI on anatomic images

**Figure 1:**
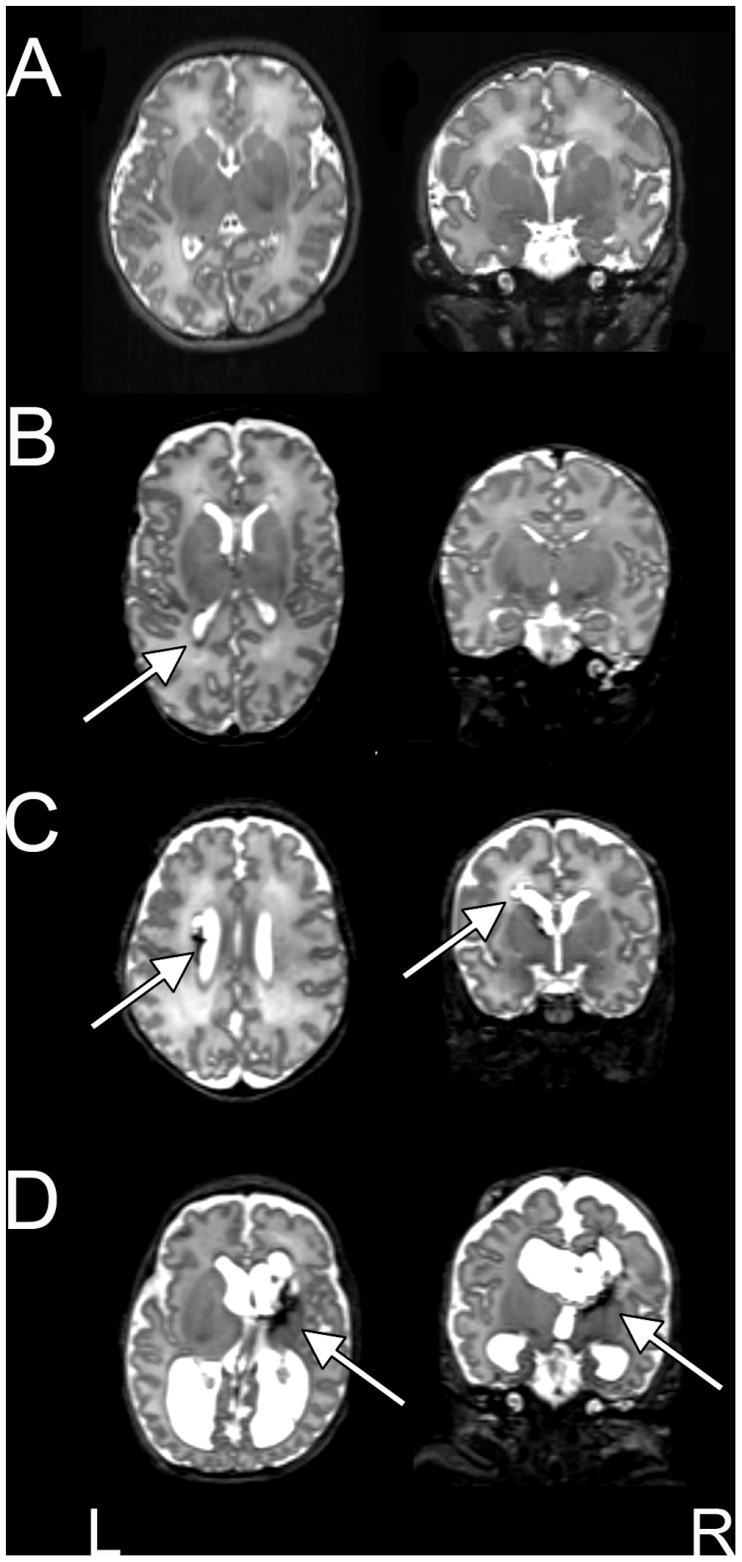
WMI in preterm infants scanned at term equivalent PMA. Transverse and coronal T2-weighted MR images illustrating (**B**) mild, (**C**) moderate and (**D**) severe WMI. Images from a healthy, term-born subject are provided for comparison (**A**). The arrows denote representative regions of injury. Areas of hemorrhage appear dark.

Anatomic MRI images were reviewed by a neuroradiologist (J.S.S.) and pediatric neurologists (C.D.S., T.E.I., J.J.N.). For all premature infants, the extent and severity of WMI was scored using a previously described system which includes criteria for presence of cystic lesions, focal signal abnormality, myelination delay, thinning of the corpus callosum, lateral ventricle dilatation and cerebral volume reduction (range 0–17) [43]. Based upon these measures, infants were categorized as having mild (WMI scores ≤5), moderate (WMI scores 6–9) or severe (WMI scores ≥10) WMI. Figure 1 provides examples of each WMI type. Cerebellar injury was also graded based upon the presence of signal abnormality and volume reduction (range 0–7) [43].

#### rs-fcMRI analysis

rs-fcMRI data were preprocessed using previously described techniques [35]. Briefly, this included correction for asynchronous slice timing and rigid body correction of head movement. Within each fMRI run, all voxels over all (magnetization steady-state) frames were multiplicatively scaled to obtain a global mode value of 1000. Such scaling enables interpretation of absolute intensity changes in terms of percentage, but has no effect on correlation computations. In addition, EPI distortions were corrected (FUGUE module in FSL [44]) using a mean field map technique [45]. Atlas transformation was computed using gestational age-specific templates [35]. Volumetric timeseries in adult Talairach atlas space (3×3×3 mm^3^ voxels) were generated by combining motion correction and atlas transformation in a single re-sampling step. Additional preprocessing in preparation for rs-fcMRI included removal by regression of nuisance waveforms derived from rigid body motion correction, regions in cerebrospinal fluid (CSF) and white matter, plus the global signal averaged over the whole brain. The data were passed through a temporal low pass filter, retaining frequencies below 0.08 Hertz, and spatially smoothed (6-mm full-width at half-maximum in each direction). Frames corrupted by motion were identified by analysis of the fully preprocessed volumetric timeseries [46] using stringent criteria (volume-to-volume head displacement 0–0.25 mm and differentiated BOLD signal intensity rms value (DVARS) <0.3%). A minimum of 100 frames (corresponding to 5 minutes) of BOLD data, after excluding frames corrupted by head motion, was required for inclusion.

Regions of interest (ROIs) were defined as 6-mm spheres in the motor, visual, auditory, posterior cingulate and medial prefrontal cortices, thalamus and medial and lateral cerebellum. For two WMI subjects, only one cerebellar ROI per hemisphere could be placed due to injury. All ROIs were initially centered upon *a priori,* atlas-derived coordinates [47]. These regions were selected based upon prior published rs-fcMRI investigations in prematurely-born infants [35]–[37]. ROI center coordinates were manually adjusted by a neuroradiologist (J.S.S.) viewing 1×1×1 mm^3^ voxel atlas space representations of T2-weighted structural images to compensate for injury-related anatomic variations. Thus, following repositioning, each individualized ROI was centered on the intended gray matter region. The repositioned ROIs were resampled to 3×3×3 mm^3^ voxel atlas space for extraction of the BOLD timeseries by averaging over all included voxels. The 6-mm ROI radius was matched to the BOLD data before blurring (6-mm full-width at half-maximum).

Correlation maps and ROI-ROI correlation coefficient matrices were computed using the standard Pearson product moment formula [20]. Correlation coefficients were Fisher *z-*transformed [48] prior to further analyses. In addition, timeseries covariance estimates were computed for selected ROI pairs. Covariance (*i.e.*, un-normalized correlation) provides a measure of temporal coherence, which, unlike Pearson correlation, retains sensitivity to the magnitude of BOLD fluctuations [49]. ROI pairs predominantly consisted of homotopic counterparts for each seed location, but also included intra-hemispheric regions (thalamus-motor cortex) and the posterior cingulate and medial prefrontal cortices (*i.e.,* midline components of the default mode network). To account for the heterogeneity in injury location within the WMI group, thalamus-motor cortex pairs were categorized as being in the hemisphere of ‘greater’ versus ‘lesser’ injury based on anatomic MR results (as opposed to right versus left). Data analysis was performed using SPSS version 19 (Chicago, IL). Correlation values were compared using a two-sample, two-tailed *t*-test. Covariance values were compared using a Mann-Whitney U two-sample rank-sum test. For these analyses, the Bonferroni multiple comparisons corrected threshold for significance level of α = 0.05 was 0.006.

## Results

### Subjects

#### Clinical information

A total of 14 preterm infants with moderate to severe WMI were evaluated. The mean gestational age at birth for the WMI group was 25.7 weeks (±2.3, range 23–29 weeks). An additional ten subjects with PHI underwent rs-fcMRI data collection, but were excluded from this analysis as they did not meet stringent rs-fcMRI data quality criteria. There were no clinical differences between subjects who were included and those who failed to meet data quality criteria. Eight infants were female and eight were white. The mean PMA at scan for these infants was 37.9 weeks (±0.9, range 36–39 weeks). The timing of scan acquisition for these subjects was determined by clinical status and medical course. The WMI cohort included ten infants with moderate and four with severe injury. Two infants had undergone ventriculoperitoneal shunt placement for post-hemorrhagic ventricular dilatation. Seven infants had cerebellar hemorrhages, six of which were punctate. The mean WMI score for these infants was 8.9 (±2.8, range 6–14). The mean cerebellar injury score for these infants was 2.7 (±1.3, range 1–6).

The preterm control group included 25 infants born prior to 30 weeks gestation with mild WMI without laterality on term equivalent anatomic MR scans. For these infants, the mean gestational age at birth was 26.8 weeks (±1.8, range 23–29 weeks). Twelve infants were female and 11 were white. The mean PMA at scan was 37.6 weeks (±1.3, range 36–40 weeks). The timing of scan acquisition for these subjects was also determined by clinical status and medical course. Five infants had cerebellar hemorrhages, four of which were punctate. The mean WMI score for these infants was 3.5 (±1.4, range 1–5). The mean cerebellar injury score for these infants was 1.1 (±1.4, range 0–5).

The term-born control group included 25 infants without cerebral injury. For this group, the mean gestational age at birth was 39.4 weeks (±1.1, range 37–41 weeks) with mean PMA at scan 39.5 weeks (±1.1, range 37–41 weeks). Sixteen infants were female and eight were white. For these subjects, scan acquisition was completed within three days following delivery. See Tables 1–3 for additional information regarding each cohort.

**Table 1:** Demographic information for preterm subjects with WMI.

Subject	Sex	Ethnicity	GA at birth (wks)	Birthweight (g)	PMA atscan (wks)	WMIScore	PHI Location	Cerebellar Injury Score	Cerebellar Hemorrhage
**wmi001**	Female	White	26	870	38	9	Unilateral, Right	2	No
**wmi002**	Female	African-American	29	1180	37	6	Unilateral, Left	3	No
**wmi003**	Female	White	29	1390	38	9	Unilateral, Right	1	No
**wmi004**	Male	White	28	1310	39	12	Bilateral, Right>Left	2	Yes – Left Punctate
**wmi005**	Female	White	25	750	37	8	Unilateral, Left	2	No
**wmi006**	Male	White	29	835	39	14	Bilateral, Right>Left	3	No
**wmi007**	Female	African-American	26	870	38	6	Unilateral, Left	1	No
**wmi008**	Male	Asian	24	700	39	9	Bilateral, Right>Left	3	Yes – Right Punctate
**wmi009**	Male	White	25	850	38	14	Bilateral, Right>Left	4	Yes – Left Punctate
**wmi010**	Female	African-American	23	610	39	10	Bilateral, Left>Right	6	Yes – Right Moderate
**wmi011**	Female	African-American	23	640	36	6	Unilateral, Right	3	Yes – Right Punctate
**wmi012**	Female	African-American	23	690	37	8	Bilateral	3	Yes – Left Punctate
**wmi013**	Male	White	26	1000	38	8	Unilateral, Right	2	No
**wmi014**	Male	White	24	1000	38	6	Unilateral, Left	3	Yes – Bilateral Punctate

Abbreviations: GA – gestational age; PMA – postmenstrual age; WMI – white matter injury; PHI – periventricular hemorrhagic infarction.

**Table 2 pone-0068098-t002:** Demographic information for preterm subjects without WMI.

Subject	Sex	Ethnicity	GA at birth (wks)	Birthweight (g)	PMA at scan (wks)	WMI Score	Cerebellar Injury Score	Cerebellar Hemorrhage
**te001**	Female	African-American	28	1155	36	2	1	No
**te002**	Female	White	28	940	38	5	1	No
**te003**	Female	African-American	26	750	40	4	1	No
**te004**	Female	White	29	1150	36	2	1	No
**te005**	Male	White	29	1523	37	2	0	No
**te006**	Male	White	25	680	36	4	3	Yes – Right Punctate
**te007**	Male	White	29	1490	36	4	0	No
**te008**	Male	White	26	770	37	5	0	No
**te009**	Male	African-American	27	1040	36	5	1	No
**te010**	Male	African-American	27	1110	39	5	2	Yes – Bilateral Punctate
**te011**	Male	African-American	27	1100	37	2	0	No
**te012**	Male	African-American	25	620	37	5	3	Yes – Bilateral Punctate
**te013**	Male	White	28	1290	37	3	1	No
**te014**	Female	White	24	800	38	5	5	Yes – Bilateral Punctate
**te015**	Female	African-American	28	920	37	2	0	No
**te016**	Male	White	29	930	40	5	1	No
**te017**	Male	White	28	1120	38	1	0	No
**te018**	Female	African-American	27	1140	39	2	0	No
**te019**	Male	African-American	27	980	37	2	0	No
**te020**	Female	African-American	25	850	37	4	4	Yes – Bilateral Moderate
**te021**	Male	African-American	23	690	39	2	0	No
**te022**	Female	African-American	24	810	39	4	0	No
**te023**	Female	African-American	25	640	37	3	3	Yes – Left Punctate
**te024**	Female	Asian	28	950	38	4	0	No
**te025**	Female	White	28	660	40	5	1	No

Abbreviations: GA – gestational age; PMA – postmenstrual age.

**Table 3:** Demographic information for term control subjects.

Subject	Sex	Ethnicity	GA at birth (wks)	Birthweight (g)	PMA at scan (wks)
**tc001**	Female	White	39	3830	39
**tc002**	Female	African-American	39	3390	39
**tc003**	Male	African-American	39	3210	40
**tc004**	Female	African-American	38	2635	39
**tc005**	Male	African-American	40	2909	41
**tc006**	Female	White	40	3320	40
**tc007**	Female	White	41	3804	41
**tc008**	Male	White	40	3702	40
**tc009**	Male	White	41	3600	41
**tc010**	Female	African-American	39	2640	39
**tc011**	Male	African-American	39	3583	39
**tc012**	Male	White	39	3033	39
**tc013**	Female	African-American	40	3195	40
**tc014**	Female	African-American	39	3230	39
**tc015**	Female	White	40	4025	40
**tc016**	Female	African-American	40	3290	40
**tc017**	Female	African-American	38	2890	38
**tc018**	Male	African-American	40	3715	40
**tc019**	Female	African-American	37	2775	38
**tc020**	Female	White	39	3230	39
**tc021**	Male	African-American	40	4054	40
**tc022**	Male	African-American	41	3205	41
**tc023**	Female	African-American	40	3685	40
**tc024**	Female	African-American	37	3060	37
**tc025**	Female	African-American	37	3230	37

Abbreviations: GA – gestational age; PMA – postmenstrual age.

#### rs-fcMRI data

WMI subjects provided an average of 156 frames (±51, range 104–279) of low-motion data (corresponding to 7.6 minutes). An average of 80 frames (34% of acquired data) was excluded due to motion (*vida supra*). There was no association between severity of WMI (p = 0.30) or cerebellar injury (p = 0.49) and number of frames removed for these subjects. An average of 147 frames (±27, range 102–188) for the term equivalent infants and 124 frames (±20, range 100–178) for the control infants were included for each group.

### Seed-Based Correlation Mapping

**Figure 2:**
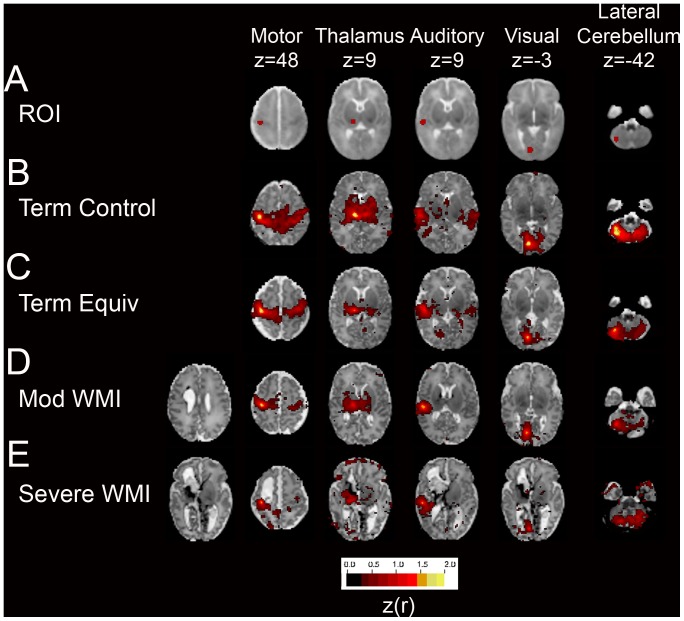
Neural network development in preterm infants with WMI scanned at term equivalent PMA. Individual rs-fcMRI correlation maps illustrating Fisher *z*-transformed correlation coefficients (z(r); color threshold value = 0.3) overlaid on the subject-specific, atlas-registered T2-weighted images. Images include (**A**) seed ROIs in the motor cortex, thalamus, auditory and visual cortices and lateral cerebellum overlaid on population-specific atlas template; (**D**) moderate and (**E**) severe WMI; (**B**) healthy, term-born subject and (**C**) preterm infant with mild WMI provided for comparison**.** For moderate-severe WMI subjects, results were generated using an ROI located in the hemisphere of greater injury. Note incomplete RSN development most prominent in the hemisphere of greater injury (always shown on figure left).

**Figure 3:**
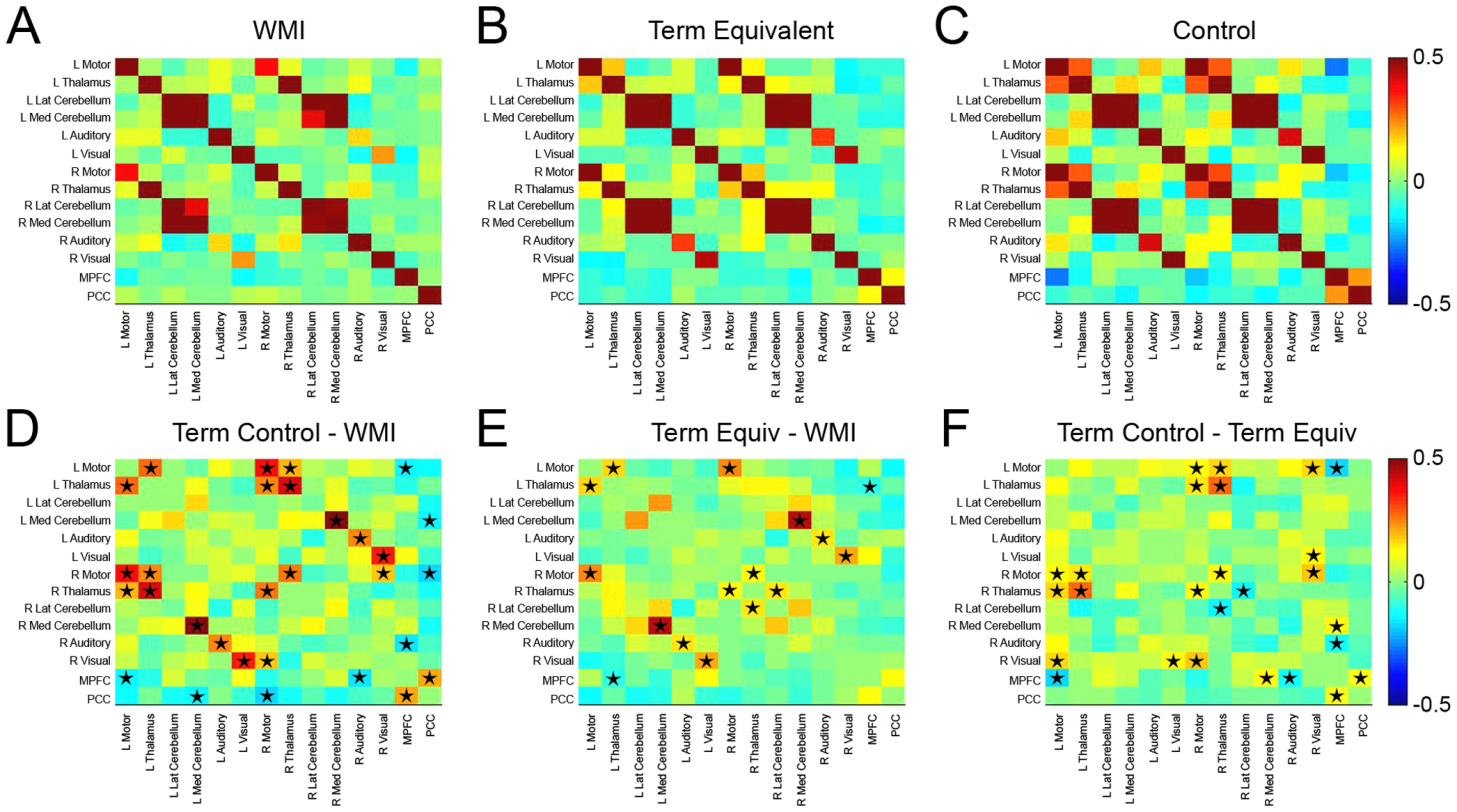
Correlation matrices. Matrices illustrating group mean Fisher z-transformed correlation coefficients for selected ROI pairs for (**A**) WMI, (**B**) term equivalent and (**C**) term control subjects. Also included are (**D**) term control – WMI, (**E**) term equivalent – WMI and (**F**) term control – term equivalent difference results. Note the lower magnitude correlation coefficients (positive as well as negative) in the WMI group in comparison to both the term equivalent and term control subjects. Black stars on matrices **D**–**F** denote cells with between group differences on two-sample, two-tailed *t*-test (p<0.05; multiple comparisons correction not performed).

**Figure 4:**
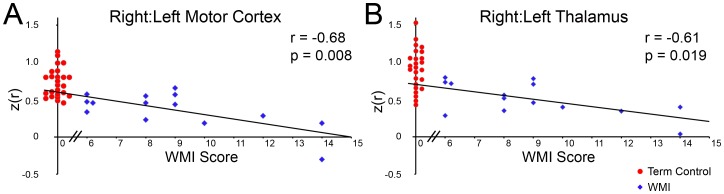
Severity of WMI correlates with loss of functional connectivity. Scatter plots demonstrating the relationship between WMI score and Fisher z-transformed correlation coefficients evaluated in homologous ROI pairs for the (**A**) motor cortex and (**B**) thalamus for WMI subjects (blue diamonds). Lines illustrate the results of z(r) on WMI score linear regression. Correlation values with significance measures included. Results for term control subjects are displayed for comparison (red circles). All term control subjects had WMI scores of 0. Symbol abscissae have been shifted to avoid overlap.

**Figure 5:**
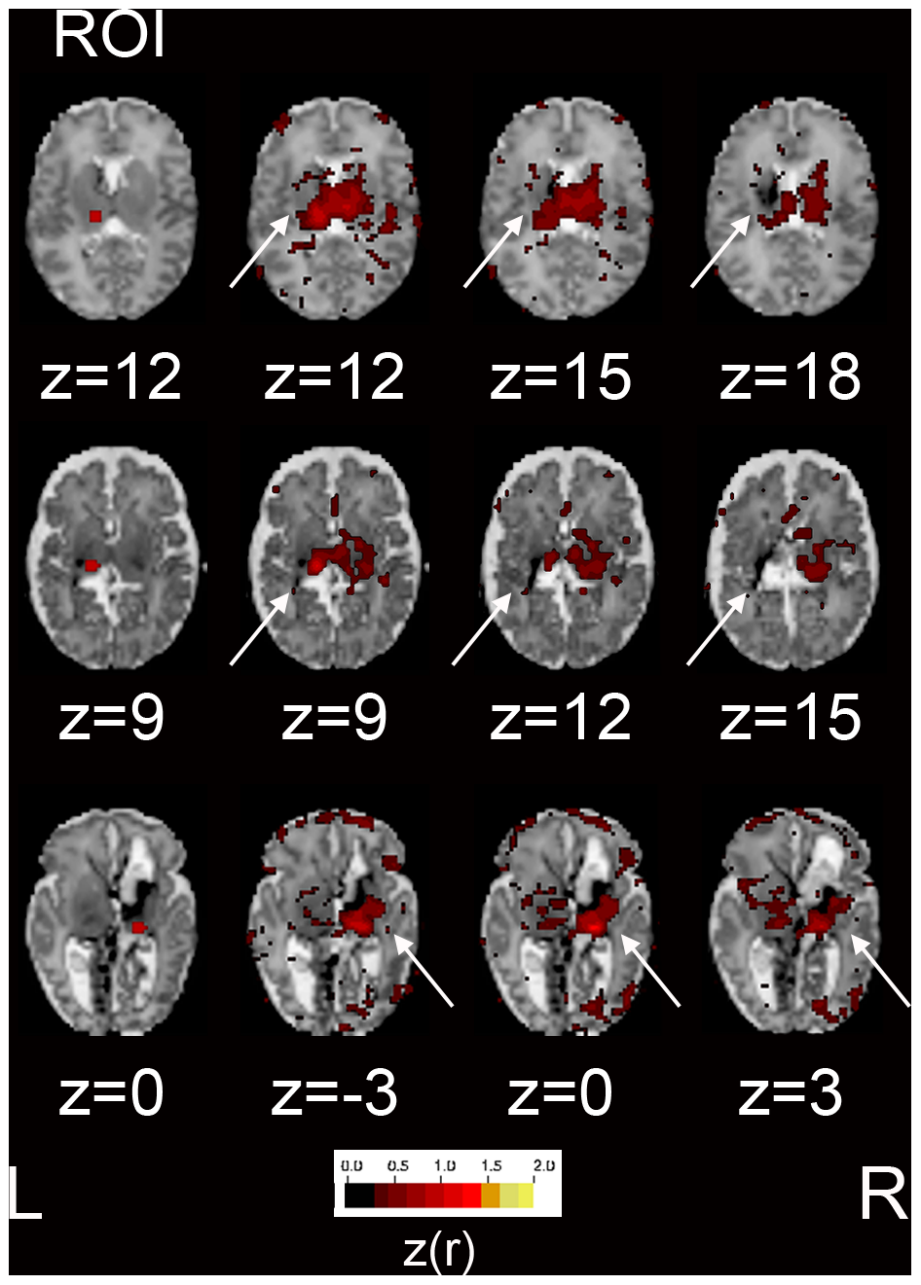
RSN topography in relation to focal injury. Fisher z-transformed correlation coefficient maps (color threshold value = 0.3) obtained with seed ROIs (illustrated in left column) overlaid on subject-specific, atlas-registered T2-weighted images. Results included from thalamic seeds in hemisphere of greater injury for infants with increasing WMI scores (scores 6, 10 and 12). Arrows denote areas of injury. Note the consistent preservation of functional connectivity in gray matter abutting the lesion and presence of functional connectivity in the hemisphere contralateral to the side of greatest injury.

RSNs in infants with moderate-severe WMI were generally similar to those observed in both control cohorts. The predominant feature in the correlation maps for all groups was inter-hemispheric symmetry indicating synchronous intrinsic fluctuations in homotopic cortical regions (Figure 2). Intra-hemispheric correlations were also present but quantitatively weaker. These findings are consistent with results previously reported in prematurely-born infants without WMI [35]–[37] and term control infants [35], [36], [38], [39]. However, infants with moderate-severe WMI showed consistent patterns of RSN abnormality:

1. BOLD signal correlations were lower in the WMI group in comparison to preterm infants without injury and term control infants. RSNs demonstrated diminished short-range (*i.e.,* ‘local bloom’ at the region of interest) and long-range (*i.e.,* between homotopic counterparts) correlation in comparison to control subjects. In infants with WMI, reduced correlations were most evident in the more injured hemisphere. Representative results are provided in Figures 2D and 2E. Measures of correlation and covariance demonstrate these patterns quantitatively (Table 2, Figure 3, Table S1 and Figure S1).
2. For the motor cortex and thalamus, the regions typically nearest to the injury site, the effect of WMI on cerebral BOLD correlations was proportional to injury severity. Infants with greater WMI scores showed more pronounced diminution in inter- and intra-hemispheric correlations across most ROI locations. These patterns are illustrated on correlation maps for infants with severe WMI (Figure 2E). These effects are also evident in scatter plots demonstrating the relationship between WMI score and correlation values for homotopic counterparts in the motor cortex (r = −0.68, p = 0.008) (Figure 4A) and thalamus (r = −0.61, p = 0.019) (Figure 4B).
3. Functional connectivity was frequently found in gray matter regions near areas of WMI, but did not extend into areas of injury (hemorrhage and/or encephalomalacia; representative examples are provided in Figure 5). In these instances, correlations with the seed region abutted, but did not include, voxels within lesions. This result suggests gray matter close to injured areas remains functionally intact.

### Quantitative ROI Pair Results

Differences between the WMI and each control group were observed for all homotopic cerebral ROI pairs (Table 4). For example, the mean Fisher z-transformed correlation values (z(r)) between right and left motor cortex ROIs were 0.36 for injured infants, 0.61 for preterm infants without WMI (p = 0.003) and 0.73 for term control infants (p<0.001). Similar patterns were seen in the thalamus and auditory and visual cortices. The combination of correlation results across homologous cortical regions (three pairs) also demonstrated differences between WMI and term equivalent (p<0.001) and term control infants (p<0.001).

**Table 4:** Mean Fisher z-transformed correlation values for WMI, term equivalent and term control infants.

ROI Pair Location	WMI	TE	Control	WMI vs Control*	WMI vs TE*	TE vs Control*
**R-L Motor Cortex**	0.36	0.61	0.73	**p<0.001**	**p = 0.003**	p = 0.033
**Motor Cortex-Thalamus Hemisphere Greater Injury**	−0.003	0.18	0.31	**p<0.001**	**p = 0.002**	p = 0.038
**Motor Cortex-Thalamus Hemisphere Lesser Injury**	0.07	N/A	N/A	**p<0.001**	p = 0.029	N/A
**R-L Thalamus**	0.51	0.64	0.92	**p<0.001**	p = 0.088	**p<0.001**
**R-L Visual Cortex**	0.23	0.44	0.58	**p<0.001**	**p<0.001**	p = 0.009
**R-L Auditory Cortex**	0.16	0.31	0.41	**p<0.001**	p = 0.027	p = 0.066
**R-L Lateral Cerebellum**	0.55	0.59	0.53	p = 0.880	p = 0.691	p = 0.549
**R-L Medial Cerebellum**	0.63	1.07	1.13	**p = 0.002**	**p = 0.004**	p = 0.579
**MPFC-PCC**	0.002	0.12	0.22	p = 0.011	p = 0.147	p = 0.028

Abbreviations: TE – term equivalent; MPFC – medial prefrontal cortex; PCC – posterior cingulate cortex.

* Result from two-sample, two-tailed *t*-test between groups; bold text denotes significant between group differences following multiple comparisons correction.

Correlations between intra-hemispheric ROI pairs and default mode network (DMN) components were also diminished in WMI subjects. For these infants, the mean z(r) value between the motor cortex and thalamus was −0.003 in the hemisphere of greater injury and 0.07 in the hemisphere of lesser injury. Each of these differed from results for preterm infants without WMI (z(r) = 0.18, p = 0.002 for greater injury hemisphere, p = 0.029 for lesser injury hemisphere) and for term subjects (z(r) = 0.31, p<0.001 for greater injury hemisphere, p<0.001 for lesser injury hemisphere). In addition, the mean correlation between the medial prefrontal and posterior cingulate cortices was 0.002 for the WMI group in comparison to 0.22 for term control infants (p = 0.011).

For medial cerebellar ROIs, the mean z(r) value of 0.63 for WMI subjects differed from term equivalent (z(r) = 1.07, p = 0.004) and term control (z(r) = 1.13, p = 0.002) results. However, correlation between left and right lateral cerebellar ROIs did not differ across groups. The presence of cerebellar hemorrhage did not affect correlation results in the cerebellum. Findings for WMI subjects with (n = 7) and without (n = 7) cerebellar hemorrhage did not differ for lateral (p = 0.66) or medial (p = 0.53) cerebellar ROI locations.

Analysis of covariance and correlation values yielded similar findings of reduced intra- and inter-hemispheric rs-fcMRI measures (Table S1 and Figure S1).

### BOLD Signal Variance

**Figure 6:**
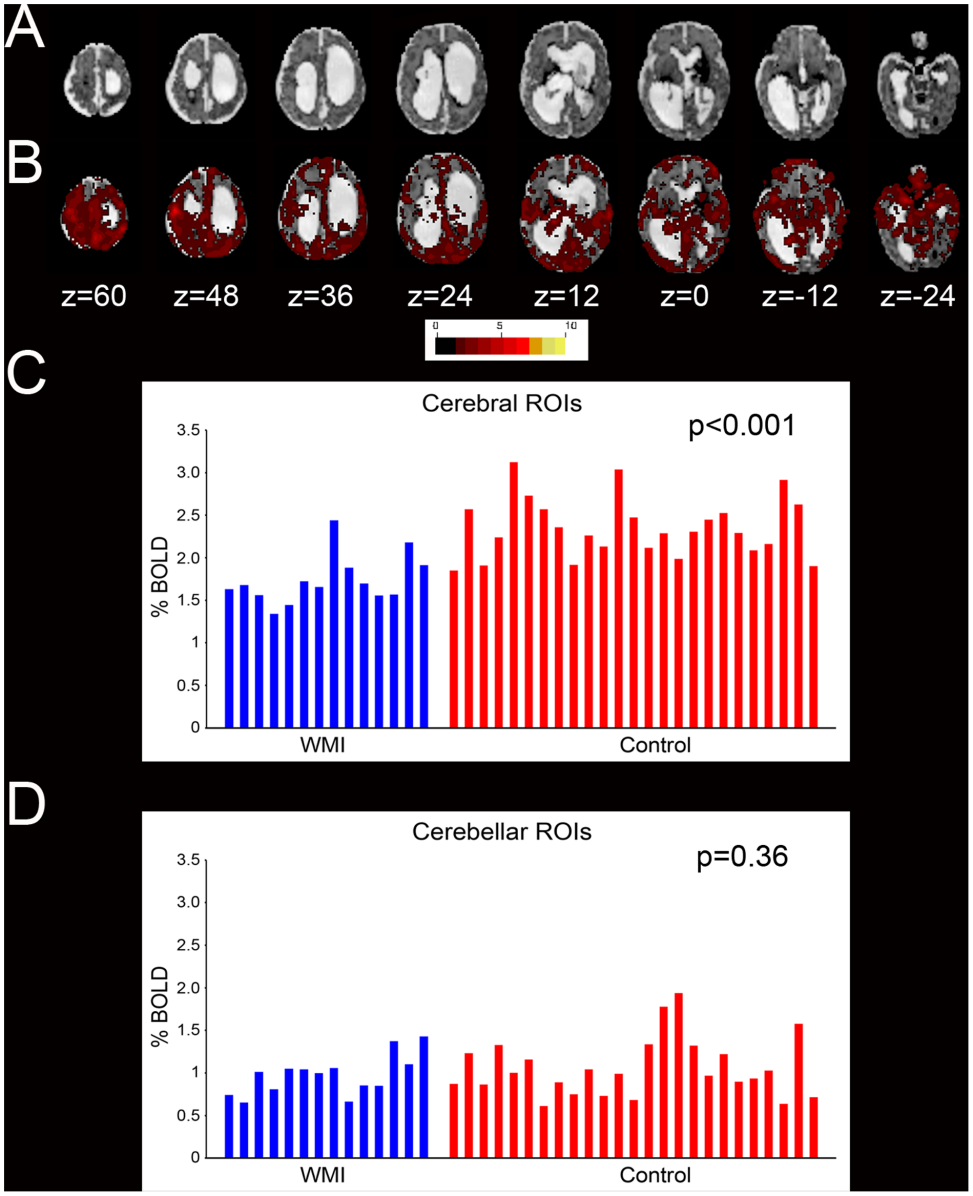
Intrinsic BOLD signal fluctuations in WMI. (**A**) T2-weighted image in an infant with severe WMI. (**B**) Voxelwise root mean squared temporal variance (signal SD; color threshold value = 0.15%). Note the paucity of activity in CSF spaces and persistence of BOLD signal fluctuations in gray matter despite severe thalamic injury. Resting state BOLD SD summed over (**C**) cerebral and (**D**) cerebellar ROIs in WMI and control infants. Each bar represents a single subject. Note the reduction of BOLD SD between the WMI (mean 1.73%) and control (mean 2.35%) groups for non-cerebellar ROIs (p<0.001) in (**C**). Values for cerebellar ROIs did not differ between WMI (mean 0.97%) and control (mean 1.06%) subjects (p = 0.36) as illustrated in (**D**). N.B., Values in panels **B**, **C** and **D** are computed relative to the whole brain mode value evaluated over all voxels and frames of each bold run.

Lower functional connectivity in the WMI group theoretically is attributable to less signal (*i.e.*, reduced amplitude of correlated intrinsic BOLD signal fluctuations) or more unstructured noise. To examine this possibility, we verified that voxel-wise BOLD signal root mean squared (rms) variance (temporal SD) was normally present within gray matter voxels as opposed to other tissues (Figure 6B). Quantitative results were obtained by computing regional BOLD signal temporal SDs summed over all ROIs, excluding those located in the cerebellum. This analysis showed lower BOLD SD values in the WMI group in comparison to term infants (p<0.001 by two-tailed *t*-test) (Figure 6C). Interestingly, the term equivalent cohort was indistinguishable from the WMI group (p = 0.89), also differing from the term control subjects (p<0.001). Additional analyses showed this result is not attributable to systematic group difference in the prevalence of head motion (excluding censored frames); moreover, regional SD was not correlated with head motion (in rms mm) evaluated over retained frames (Figure S2). Results from analysis including only ROIs located in the cerebellum demonstrated no SD differences between WMI and term control (p = 0.36) (Figure 6D) or term equivalent (p = 0.45) cohorts.

## Discussion

Despite significant advances in neonatal and perinatal care, premature infants remain at great risk for cerebral injury, with particular vulnerability in the cerebral white matter. This susceptibility of immature white matter results from a confluence of maturation-dependent factors, with ischemia and systemic inflammation playing important roles in injury pathogenesis [3], [4]. Multiple risk factors for WMI have been identified, including degree of prematurity, maternal chorioamnionitis, sepsis, hypotension, hypocarbia, need for inotropic support, pneumothorax, postnatal corticosteroids and patent ductus arteriosus [3], [50]. More importantly, the adverse impact of moderate-severe WMI on motor, sensory and cognitive function is established, though considerable variability exists in its neurodevelopmental effects [4].

Previous MR studies of infants, children, adolescents and young adults born preterm have used advanced neuroimaging modalities, principally DTI, to characterize structural and functional alterations in brain development related to WMI [51]. These investigations demonstrated microstructural abnormalities in premature infants with WMI that correlated with injury severity and early outcome [11], [12], [14], [19], [52]. Investigations during later developmental epochs suggest that these abnormalities persist [15], [18] and correlate with functional performance measures [16].

More recent investigations of older subjects with perinatal WMI have also included rs-fcMRI. Lee and colleagues demonstrated diminished RSN strength in motor regions of children, adolescents and young adults with spastic diplegia due to WMI and correlated these findings with severity of functional impairment [34]. Burton and colleagues also identified aberrant functional connectivity in motor regions in young adults with spastic diplegia [33]. Finally, Wingert and colleagues demonstrated diminished cortical activation in response to sensory stimulation on task-based fMRI during adolescence and early adulthood in subjects with cerebral palsy [32]. Our investigation suggests that these effects are identifiable early in development, which is consistent with complementary (*e.g.,* DTI) imaging studies.

rs-fcMRI is well established as a technique for assessing the functional integrity of the brain in a variety of adult neurological and psychiatric entities [53], though it has had limited application to the study of sick or injured infants. We have previously shown that prematurity is associated with subtle rs-fcMRI changes by term equivalent PMA in infants without overt WMI, a finding corroborated in this investigation [35]. In this study, we demonstrate more pronounced rs-fcMRI abnormalities in infants with moderate-severe WMI. Further, in regions nearby the site of injury, quantitative rs-fcMRI measures negatively correlate with lesion severity. The finding of reduced inter-hemispheric functional connectivity in infants with WMI is concordant with previous studies. In adults, inter-hemispheric functional connectivity averaged over several homologous cortical regions was reduced in proportion to corticospinal tract injury due to white matter strokes, and correlated with motor performance measures [54]. In addition, results in adults indicate that reduced inter-hemispheric functional connectivity is a marker of impaired awareness [55].

We observed rs-fcMRI group differences for medial, but not lateral, cerebellar ROIs. These networks likely are early forms of RSNs incorporating cortical and cerebellar regions that have been identified in older populations [56]–[60]. Similar cerebellar RSNs have been reported in term and preterm subjects [35]–[37], though with some inconsistencies with respect to cerebellar findings, *i.e.,* RSNs present [35]–[37] or absent [38]. The abnormalities we detected may be related to injury to the cerebral hemispheres, as a growing body of literature has correlated the effects of supratentorial brain injury with cerebellar development [13], [61]. Further, abnormalities of cerebellar RSNs may have clinical consequences, as cerebellar injury has been shown to affect supratentorial brain development and correlate with early childhood outcomes [62], [63]. Additional investigation of these effects in subjects with and without supratentorial and cerebellar injury remains necessary.

We also found that the BOLD signal SDs summed over cerebral ROIs (Figure 6C) were lower in the WMI and term equivalent groups. fMRI findings of this type are conventionally reported as reduced amplitude low frequency fluctuations (ALFF). Reduced ALFF has been observed in a wide spectrum of neurologic and psychiatric disorders [49], [64]–[66]. Several infants with severe WMI also sustained structural damage in subcortical structures (*i.e.,* thalamus), raising the possibility that injury to these regions underlies suppressed intrinsic activity. In these infants, reduced ALFF might be predicted as a consequence of interrupted cortical-striatal-thalamic loops [67]. The pathophysiological basis of low ALFF in infants with WMI requires further study.

Anatomical and functional connectivity are interrelated, but not identical [68], [69]. The effects of WMI on RSN development are likely multifactorial, reflecting the complex evolution of anatomic infrastructure that underlies early functional brain development. The destructive and maturational sequelae of WMI have been extensively detailed [4], [70], [71]. WMI engenders pattern-specific local and widespread effects (*e.g.,* deafferentiation) as well as immediate and delayed consequences [72]. Direct effects of WMI include loss of premyelinating oligodendrocytes [73], [74], axonal damage and gliosis [75], [76] with consequent delayed or aberrant myelination. These changes may be focal or diffuse [77] and evolve over time [75]. WMI has also been increasingly recognized to influence cortical, subcortical and cerebellar gray matter development [4]. The resulting neuronal loss and gliosis preferentially affects vulnerable regions, including specific cortical layers [70] and critical neurodevelopmental areas including the subventricular and subplate zones [71]. The impact of this neuronal injury on neurodevelopment typically depends upon the location and degree of WMI, with severely affected infants manifesting more prominent and widespread sequelae from injury [4]. The present data suggest that rs-fcMRI measures also reflect these effects. However, the persistence of RSNs, despite moderate-severe WMI, underscores the resiliency and potential of early functional brain development.

### Several Caveats and Limitations Affect the Present Results

1. *Sample Size.* Due to rigorous entry and data quality criteria, this investigation included a total of 14 neonates with moderate-severe WMI, 25 term equivalent and 25 term control infants. While this is a relatively small sample, quality assurance is critical to obtaining accurate data (*vide infra*). Among the group with WMI, there was diversity in the injury patterns, representing the heterogeneity inherent to WMI. This limited and mixed population restricted our ability to perform some investigations previously implemented in this population (such as analysis of group mean correlation maps). Additional group-level associations may become feasible with larger samples of subjects demonstrating more homogenous WMI patterns.
2. *rs-fcMRI Data Acquisition.* rs-fcMRI technique can affect results obtained in subjects of any age. The acquisition parameters and analysis methods were selected to maintain consistency with prior investigations in this population and have been utilized to perform more than 200 rs-fcMRI acquisitions in neonates. Further systematic study of the effects of these variables and establishment of optimal acquisition parameters for rs-fcMRI data in neonates remains necessary.
3. *rs-fcMRI Data Analysis.* Analytic methodology affects results. The advantages and limitations of seed correlation analysis (SCA) have been extensively discussed [26], [27], [78]–[80]. Due to the anatomic heterogeneity in infants with WMI, we performed SCA using ROIs individualized for each subject. This approach was designed to maximize anatomic specificity. It has been suggested that cm-scale variability in seed ROI location minimally affects BOLD correlation results obtained in anatomically intact adults [81]. Indeed, our results at the group level were minimally affected by the manual adjustment of ROI placement (results not shown). However, as illustrated in Figure 5, it is important in studies of the present design to avoid evaluating rs-fcMRI in injured parenchyma. Future inquiries expanding the number of seed locations may provide greater understanding of the effects of WMI.
4. *Motion.* Recent reports have documented that rs-fcMRI is exquisitely sensitive to subject motion [35], [46]. Neonates constitute a population predisposed to motion during rs-fcMRI acquisition. Therefore, we employed meticulous frame censoring (“scrubbing”) to minimize the impact of motion-related artifacts. Further investigation may reveal better approaches for identifying the colored noise introduced by motion into rs-fcMRI data and the measures necessary to account for its presence.

### Conclusions

To date, neuroimaging investigations of WMI in infants have demonstrated only modest correlation with neurodevelopmental outcomes, and the neural mechanisms leading to these impairments remain incompletely understood. One potential means of improving both our understanding of these mechanisms and the prognostic accuracy of neuroimaging is to incorporate information concerning RSN integrity obtained via rs-fcMRI. This report illustrates the feasibility of applying rs-fcMRI to investigate effects on RSNs associated with WMI in prematurely-born infants. We demonstrate regionally-specific effects that correlate with injury severity in a heterogeneous sample. These results suggest future rs-fcMRI investigations have the potential to provide greater understanding of the mechanisms underlying adverse neurodevelopmental outcomes and to improve the prognostic accuracy of neuroimaging studies.

## Supporting Information

Figure S1 **Covariance matrices.** Matrices illustrating group mean covariance values for selected ROI pairs for **(A)** WMI, **(B)** term equivalent and **(C)** term control subjects. Also included are **(D)** term control – WMI, **(E)** term equivalent – WMI and **(F)** term control – term equivalent difference results. Note the lower magnitude correlation coefficients (positive as well as negative) in the WMI group in comparison to both the term equivalent and term control subjects. Black stars on matrices **D**–**F** denote cells with between group differences on Mann-Whitney U two-sample rank-sum test (p<0.05; multiple comparisons correction not performed).
(TIF)

Figure S2 **BOLD signal variance and head motion are not correlated.** Scatter plot demonstrating the relationship between rms head motion values and SD measures over all ROIs for term control (red circles), term equivalent (green squares) and WMI (blue diamonds) subjects. Line illustrates the results of SD on rms linear regression across all subjects. Note the limited relationship between SD and rms values.
(TIF)

Table S1 **Mean covariance values for WMI, term equivalent and term control infants.**
(DOCX)
