# Supplementary figures and images for: Effects of White Matter Injury on Resting State fMRI Measures in Prematurely Born Infants
Source: PLoS One. 2013 Jul 9;8(7):e68098. doi: 10.1371/journal.pone.0068098 (PMC3706620; doi:10.1371/journal.pone.0068098)

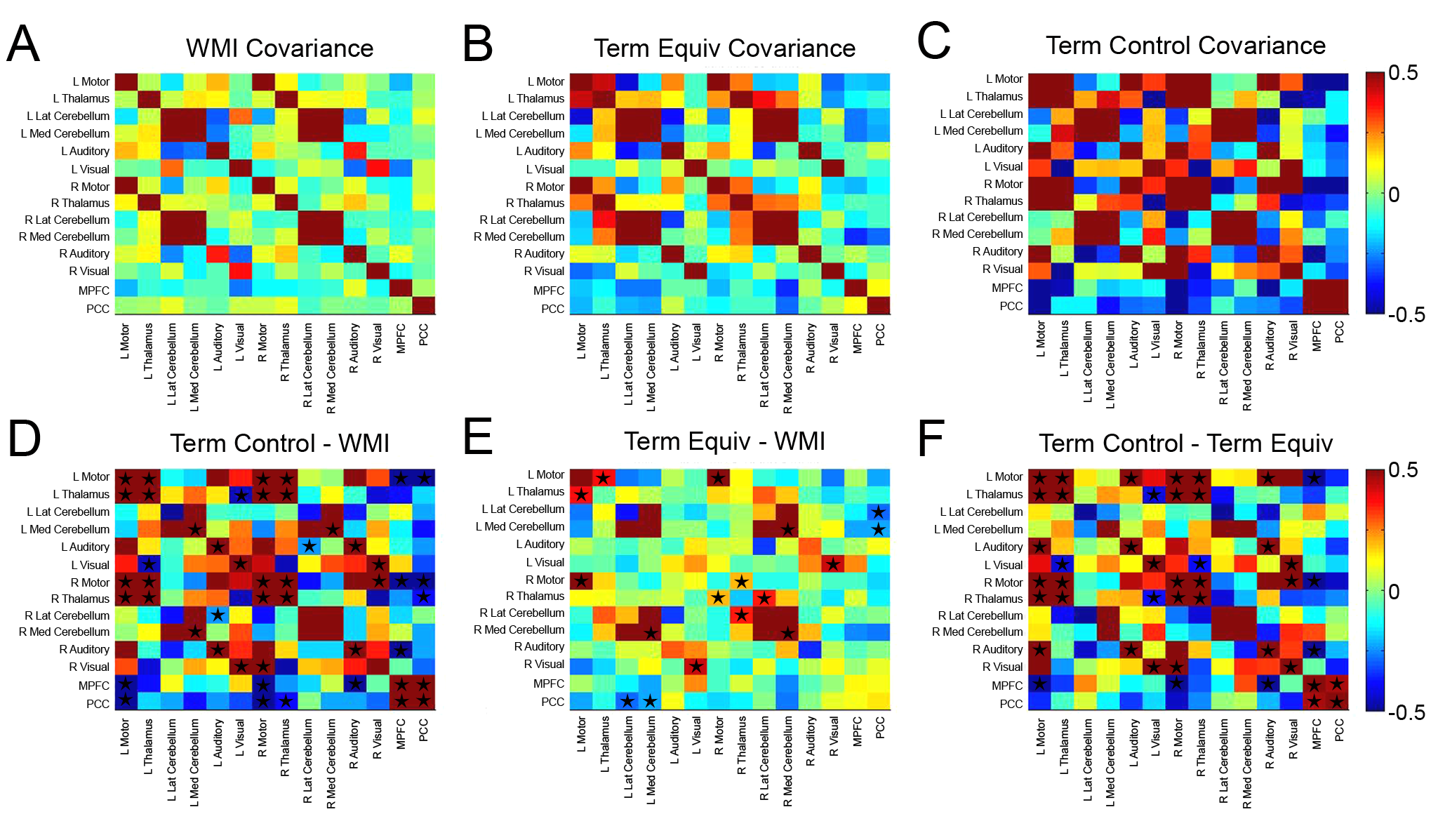

Supplement: Figure S1 — Covariance matrices. Matrices illustrating group mean covariance values for selected ROI pairs for (A) WMI, (B) term equivalent and (C) term control subjects. Also included are (D) term control – WMI, (E) term equivalent – WMI and (F) term control – term equivalent difference results. Note the lower magnitude correlation coefficients (positive as well as negative) in the WMI group in comparison to both the term equivalent and term control subjects. Black stars on matrices D–F denote cells with between group differences on Mann-Whitney U two-sample rank-sum test (p<0.05; multiple comparisons correction not performed). (TIF) [file pone.0068098.s001.tif]

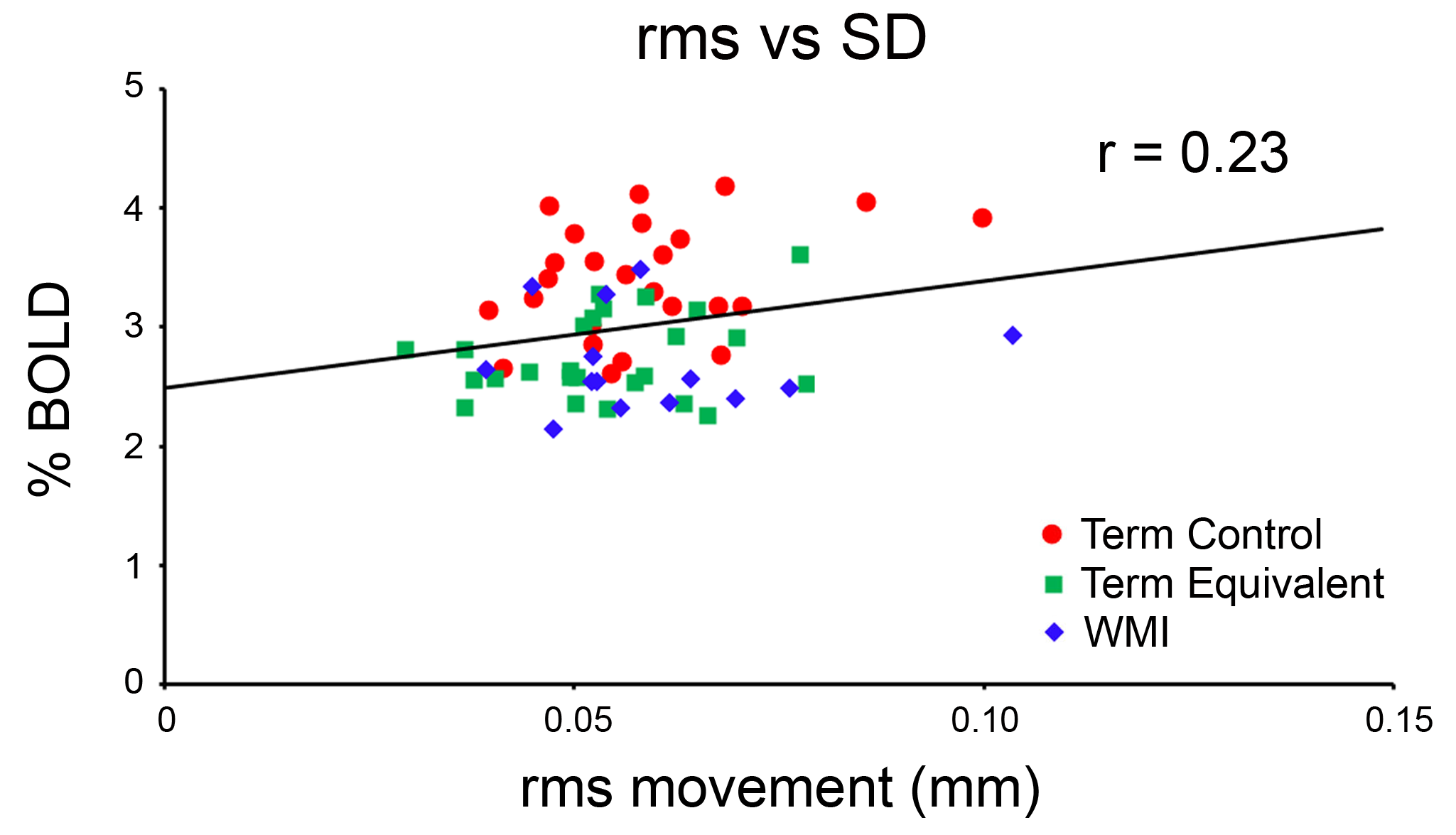

Supplement: Figure S2 — BOLD signal variance and head motion are not correlated. Scatter plot demonstrating the relationship between rms head motion values and SD measures over all ROIs for term control (red circles), term equivalent (green squares) and WMI (blue diamonds) subjects. Line illustrates the results of SD on rms linear regression across all subjects. Note the limited relationship between SD and rms values. (TIF) [file pone.0068098.s002.tif]
