# Supplementary material for: Effects of White Matter Injury on Resting State fMRI Measures in Prematurely Born Infants
Source: PLoS One. 2013 Jul 9;8(7):e68098. doi: 10.1371/journal.pone.0068098 (PMC3706620; doi:10.1371/journal.pone.0068098)
Supplement: Table S1 — Mean covariance values for WMI, term equivalent and term control infants. (DOCX) [file pone.0068098.s003.docx]

**Table S1. Mean covariance values for WMI, term equivalent and term control infants.**

| **ROI Pair Location** | **WMI** | **TE** | **Control** | **WMI vs Control*** | **WMI vs TE*** | **TE vs Control*** |
| --- | --- | --- | --- | --- | --- | --- |
| **R-L Motor Cortex** | 0.88 | 1.61 | 3.15 | **p<0.001** | p=0.020 | **p<0.001** |
| **Motor Cortex-Thalamus Hemisphere Greater Injury** | 0.01 | 0.42 | 1.27 | **p<0.001** | **p<0.001** | **p=0.002** |
| **Motor Cortex-Thalamus Hemisphere Lesser Injury** | 0.12 | N/A | N/A | **p<0.001** | p=0.009 | N/A |
| **R-L Thalamus** | 0.67 | 0.77 | 2.51 | **p<0.001** | p=0.496 | **p<0.001** |
| **R-L Visual Cortex** | 0.37 | 0.79 | 2.45 | **p<0.001** | p=0.012 | **p<0.001** |
| **R-L Auditory Cortex** | 0.35 | 0.65 | 1.79 | **p<0.001** | p=0.051 | **p<0.001** |
| **R-L Lateral Cerebellum** | 2.48 | 2.67 | 2.10 | p=0.828 | p=0.942 | p=0.635 |
| **R-L Medial Cerebellum** | 1.67 | 4.26 | 5.80 | **p=0.003** | **p=0.002** | p=0.854 |
| **MPFC-PCC** | 0.02 | 0.12 | 0.58 | **p<0.001** | p=0.185 | **p=0.001** |

Abbreviations: TE – term equivalent; MPFC – medial prefrontal cortex; PCC – posterior cingulate cortex

*Result from Mann-Whitney U two-sample rank-sum test; bold text denotes significant between group differences following multiple comparisons correction
